# Physical training in boys with Duchenne Muscular Dystrophy: the protocol of the No Use is Disuse study

**DOI:** 10.1186/1471-2431-10-55

**Published:** 2010-08-06

**Authors:** Merel Jansen, Imelda JM de Groot, Nens van Alfen, Alexander CH Geurts

**Affiliations:** 1 Radboud University Nijmegen Medical Centre, Nijmegen Centre for Evidence Based Practice, Department of Rehabilitation, Nijmegen, the Netherlands; 2 Radboud University Nijmegen Medical Centre, Department of Neurology, Nijmegen, the Netherlands

## Abstract

**Background:**

"Use it or lose it" is a well known saying which is applicable to boys with Duchenne Muscular Dystrophy (DMD). Besides the direct effects of the muscular dystrophy, the increasing effort to perform activities, the fear of falling and the use of personal aids indirectly impair leg and arm functions as a result of disuse. Physical training could oppose this secondary physical deterioration. The No Use is Disuse (NUD) study is the first study in human subjects with DMD that will examine whether a low-intensity physical training is beneficial in terms of preservation of muscle endurance and functional abilities. The study consists of two training intervention studies: study 1 "Dynamic leg and arm training for ambulant and recently wheelchair-dependent boys with DMD and, study 2 "Functional training with arm support for boys with DMD who have been confined to a wheelchair for several years". This paper describes the hypotheses and methods of the NUD study.

**Methods:**

Study 1 is an explorative randomized controlled trial with multiple baseline measurements. Thirty boys with a DNA-established diagnosis of DMD will be included. The intervention consists of a six-months physical training during which boys train their legs and arms with active and/or assisted cycling training equipment. The primary study outcomes are muscle endurance and functional abilities, assessed with a Six-Minute Bicycle Test and the Motor Function Measure. Study 2 has a within-group repeated measurements design and will include ten boys with DMD who have already been confined to a wheelchair for several years. The six-months physical training program consists of 1) a computer-assisted training and 2) a functional training with an arm support. The primary study outcome is functional abilities of the upper extremity, assessed with the Action Research Arm Test.

**Discussion:**

The NUD study will fill part of the gap in the current knowledge about the possible effects of training in boys with DMD and will increase insight into what type of exercise should be recommended to boys with DMD. The study will finish at the end of 2010 and results are expected in 2011.

**Trial registration:**

The Netherlands National Trial Register1631

## Background

Duchenne Muscular Dystrophy (DMD) is an inherited X chromosome-linked recessive myopathy which affects approximately 1/4200 live-born boys[1]. DMD is characterized by a total, or near-total (<3%), absence of the cell membrane protein dystrophin. The absence of dystrophin results in a steady degradation of muscle fibers that causes progressive loss of muscle strength and functional abilities[2,3]. Boys with DMD are usually confined to a wheelchair at the age of ten years[4] and have a median life expectancy of thirty years with spinal surgery and assisted ventilation[5]. Although ongoing studies show promising therapies that target disease cause, there is still no curative (pharmaco)therapy available and, thus, treatment remains symptomatic. An important aim in the management of boys with DMD is to preserve functional abilities for as long as possible[6]. Delaying the loss of functional abilities is relevant for all activities in daily life and may optimize independence in boys with DMD.

The loss of functional abilities is primarily the result of a progressive decrease in muscle strength and muscle endurance during the course of the disease[4,7]However, increasingly limited physical and social possibilities gradually cause a secondary reduction of physical activity. Indeed, the increasing amount of energy a certain activity costs, the increasing frequency of falling (with the need for help to stand up), and the developing fear of falling further reduce leg and arm activities, resulting in disuse of the musculoskeletal and cardiorespiratory systems[8]. The use of an electrical wheelchair limits arm functions (like reaching and lifting) even more, since a top blade and a central operating joy stick force boys to function within the confines of the wheelchair. As the increased sedentary lifestyle results in progressive disuse, secondary physical deterioration will occur in all boys with DMD. Disuse in DMD thus can be defined as the discrepancy between a boy's potential capacity and his actual performance. To underscore the importance of disuse, previous studies have shown that the presence of hip, knee and elbow flexion contractures is strongly related to the onset of wheelchair dependence[4]. Another example is that boys with DMD have a higher risk of bone fractures due to osteoporosis caused by unloading[9]. Fractures as a result of falling are followed by a loss of ambulation in 20-40 percent of the cases[10,11]. In these aspects, the well-known saying "use it or lose it" is certainly applicable to boys with DMD.

Physical training could oppose the secondary deterioration of muscle tissue and the loss of functional abilities as a result of disuse. However, the number of studies that examined the effects of training in DMD is limited, and only a few training studies are reported in human subjects with DMD. These studies focused on resisted exercises in ambulatory boys and concluded that sub-maximal resistance exercises had only limited positive effects on muscle strength and timed functional tests (such as the time it takes to walk 23 feet) but, importantly, they did not cause physical deterioration[12-14]. Recent studies in mdx mice (an animal model for DMD) concluded that voluntary wheel running (dynamic training) had positive effects on muscle strength[15] and fatigue resistance[15-17]. In addition, non-weight bearing low-intensity exercises (like swimming) had no detrimental effects in mdx mice[18]. However, extrapolating data from animal studies to humans should only be done with great caution because of differences in phenotypic expression and biomechanical differences between humans subjects and animal models with muscular dystrophy[19]. Based on the currently available evidence, and clinical experience, international guidelines recommend ambulatory boys to perform voluntary (eventually mechanically-assisted) active exercises (such as swimming) and to avoid eccentric exercises. Non-ambulant boys are advised to perform mobilizing passive or actively-assisted mobilizing exercises to maintain postural symmetry and sitting comfort[20].

The mechanisms by which training may oppose the deterioration of muscle tissue is unclear. Muscle fibres in DMD are abnormally vulnerable to contraction-induced injury due to the absence of mechanical reinforcement of the sarcolemmal membrane[21]. Therefore, eccentric exercises should be avoided[22]. On the other hand, work-induced damage can enhance muscle regeneration and repair[23], and low-stress exercise may produce beneficial effects on myofiber contractility and energetic efficiency[21]. For example, low-intensity training decreased oxidative stress markers[24] and caused a shift from fast-twitch muscle fiber type 2 to slow-twitch muscle fiber type 1 in mdx mice[16]. Slow-twitch muscle fibers are more resistant to muscle fiber degeneration[16,25]. In addition, corticosteroids could support the beneficial effect of training in DMD, since steroids may prevent post-exercise deterioration of skeletal muscle[22].

**Figure 1:**
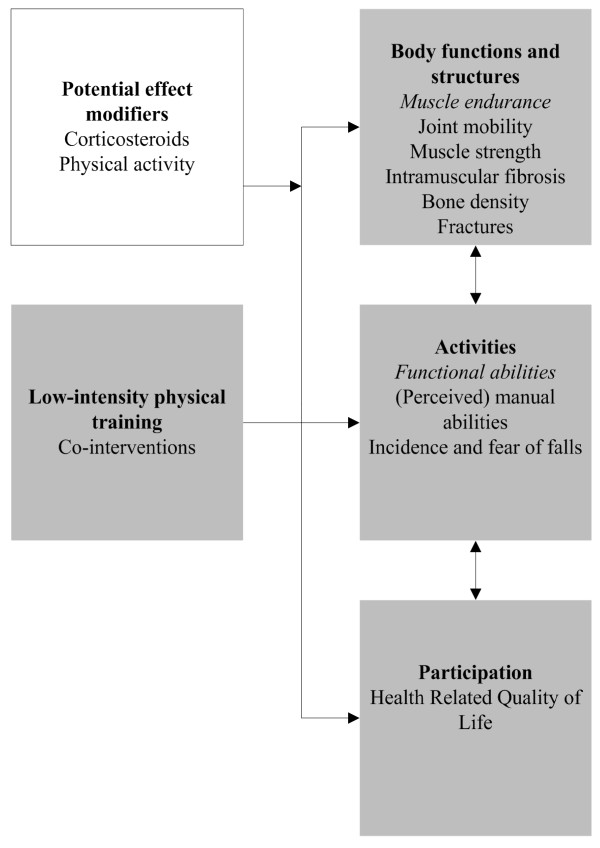
Hypotheses

The currently recommended voluntary exercises to maintain comfort and symmetry[6] are widely used in daily practice and assume that low-intensity training is beneficial and not harmful for boys with DMD. However, these recommendations are mainly based on theory and there is a need for more research to justify these recommendations and to define the optimal exercise programmes[26]. As we expect that a low-intensity training may slow-down the secondary decline of muscle endurance and functional abilities (figure 1), we developed the *No Use is Disuse *(NUD) study protocol to answer the clinically relevant questions whether low-intensity physical training in boys with DMD is beneficial and does not cause further harm. The NUD study is the first to examine the effects of low-intensity physical training in human subject with DMD and will fill part of the gap in current knowledge about the possible effects of training in boys with DMD. The study consists of two training intervention studies: study 1 "Dynamic leg and arm training for ambulant and recently wheelchair-dependent boys with DMD and, study 2 "Functional training with arm support for boys with DMD who have been confined to a wheelchair already for several years". This paper describes the hypothesis and methods of the NUD study.

## Methods

### Study 1 Dynamic leg and arm training

#### Design

Study 1 is an explorative randomized controlled trial with multiple baseline measurements. Randomization is stratified according to the ambulatory status (ambulant/non-ambulant) of the boys and the boys are allocated to the intervention group or the control group in a 2:1 ratio. The intervention group receives dynamic exercise treatment, whereas the control group receives usual care (no specific intervention) during 24 weeks. After this period, the control group will also receive the physical training. Randomization is performed by an independent statistician. Despite randomization, differences in baseline characteristics between the intervention group and the control group may still be present due to the relatively small number of participants. For this reason, multiple baseline measurements are performed that will allow us to do within-subject analyses as well. Assessments are not blinded, because it was considered virtually impossible to prevent the boys from revealing their group allocation to the assessor.

#### Participants

**Figure 2:**
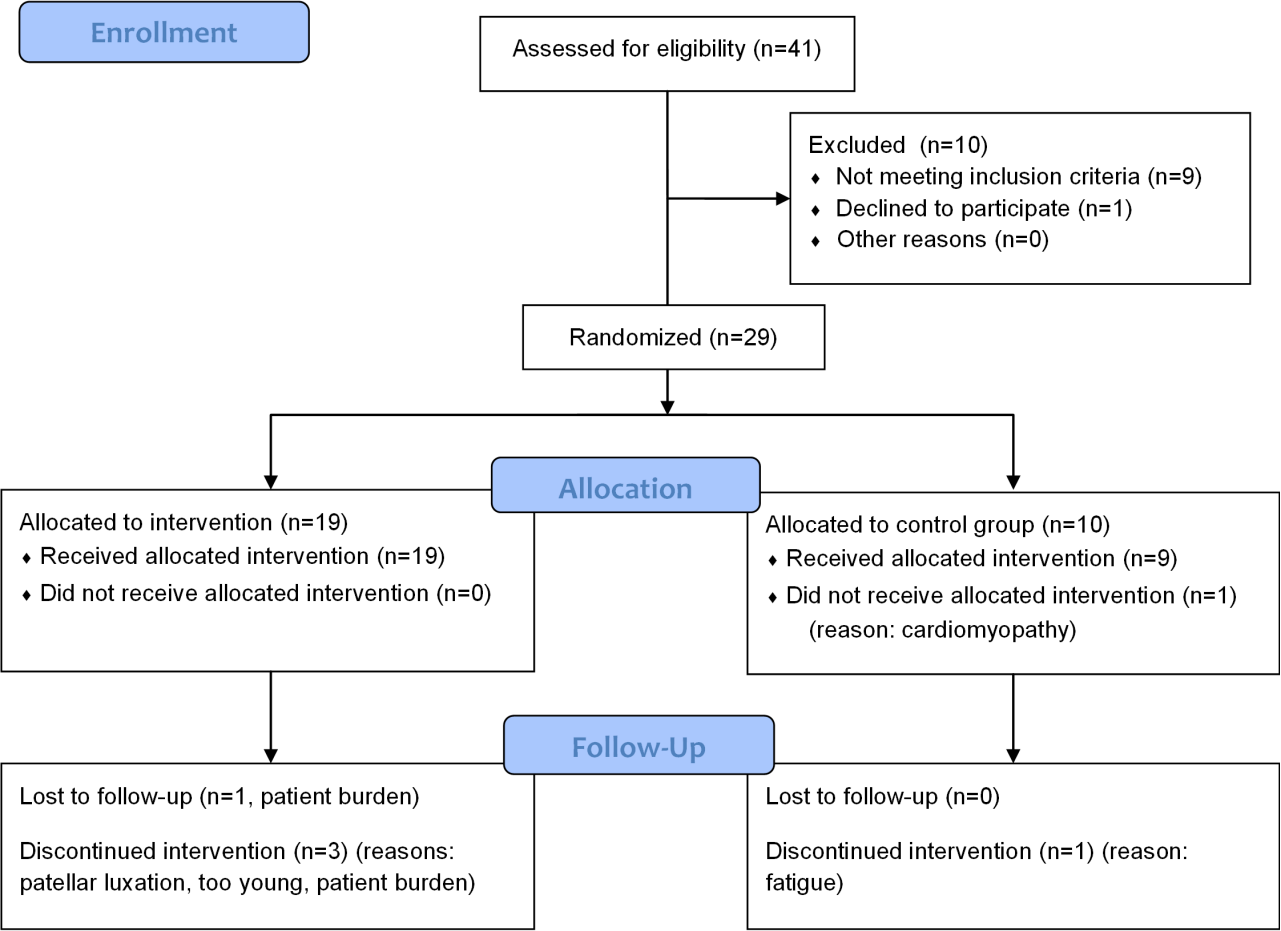
**Progress study 1 Dynamic leg and arm training**. This flowchart is a preliminary version. Final numbers may change depending on eligibility as recruitment is not yet complete.

Thirty ambulatory or recently wheelchair-dependent boys with a DNA-established diagnosis of DMD will be included (figure 2). There are an estimated four hundred boys with DMD in the Netherlands. Inclusion and exclusion criteria are described in table 1. Participants will be recruited from the Dutch Duchenne Parent Project (DPP) database. Members of the DPP will receive written information and a registration form. Additionally, we will place an advertisement on the website of the Vereniging Spierziekten Nederland (VSN), and rehabilitation physicians affiliated with the VSN will be asked to make potential participants aware of the NUD study. After registration, interested potential participants are visited by the primary investigator (MJ) at home for providing further information. Parents, and participants who are over 12 years of age, need to give written informed consent. The study protocol was approved by the Medical Ethics Committee Arnhem-Nijmegen, the Netherlands.

**Table 1:** Inclusion and exclusion criteria study 1

Inclusion criteria	Exclusion criteria
A DNA-established diagnosis of DMD	Other disabling diseases influencing mobility
Boys who are at the end of their ambulation phase, and: -need ≥5 sec. to get up from the floor or -are not able to get up from the floor or -are not able to bicycle without assistance -are dependent on a wheelchair to move over a long distance (>500 m)	Boys with a clinical symptomatic cardiomyopathy Boys <6 years old
Boys who recently became wheelchair-dependent (approximately 1-2 years after they stopped walking), and: -are able to touch the top of their head with both hands without assistance or -are not able to bicycle without assistance -are able to use a hand-operated wheelchair	

#### Intervention: dynamic leg and arm training

Boys train their legs and arms with bicycle training equipment (KPT Cycla, Kinetic, France). As this type of training equipment can be used actively or with electrical motor support, it allows a combination of active and passive bicycle training. Physical training is additional to any regular therapy or daily physical activity that boys already take part in, yet all possible co-interventions are registered.

**Figure 3:**
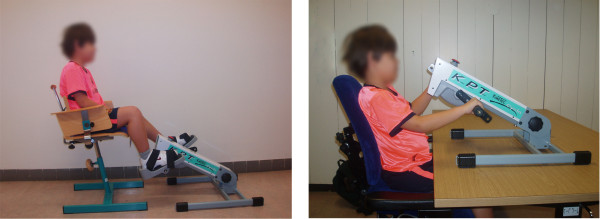
Posture during dynamic leg and arm training

Boys train at home or at school (depending on their preferences) during 30-min sessions (15 min leg and 15 min arm training), five days per week during 24 weeks. They are instructed to cycle with a continuous speed and stimulated to reach 700-1000 revolutions with both their legs and arms during each training session without getting too tired. Cycling characteristics are standardized (figure 3) and crank-arm length is adjusted to the child's height (e.g. a short crank-arm length for small legs and arms). Boys are free to choose when they would like to train, their training sequence (first arms or legs) and whether they want a break between the two 15 min training sessions or not. However, these training agreements are decided beforehand and registered in a training contract to which they should adhere. Additionally, the boys are recommended to train while watching television to make the training more pleasurable.

Based on general training principles and based on the results from previous training studies in boys with DMD and in animal models of DMD, the training intensity is low to moderate. Intensity is determined by an adjusted and modified Six-Minute Bicycle ergometer Test (see study outcomes). Boys are encouraged to reach as many revolutions as possible during this sub-maximal test. The number of revolutions is an indication for training intensity (level of electrical assistance). After six minutes of rest, boys cycle for three more minutes at the indicated training intensity. During this period, they should be able to cycle with a continuous speed of 60 revolutions per minute (RPM) with a perceived level of exertion ranging from 'a little tired' to 'getting more tired', as assessed with the OMNI Scale of perceived exertion[27]. The primary investigator will check the training intensity once again during a 15 min training session within two weeks after the start of the intervention period. This control is carried out in a home setting, so that training posture can be checked as well. When boys are not able to bicycle at a continuous speed of approximately 60 RPM without having pain or maintaining the level of 'getting more tired', the training intensity will be lowered.

As described above, training intensity is based on the ability to bicycle at a continuous speed and perceived exertion. Intensity is, thus, not based on peak heart rate, which is common in the literature on physical exercise training. The reason for this is twofold. Firstly, boys with DMD have a higher resting heart rate (110 ± 12 beats/min) compared to healthy controls (94 ± 7 beats/min )[28]. Additionally, boys with DMD are often forced to terminate a bicycle ergometry test while their peak heart rate is only 120-130 beats/min, since the main limiting factor during ergometry seems to be not their oxygen transport but their 'peripheral' capacity (muscle endurance, anaerobic power and muscle strength)[7].

Boys are instructed to send their daily number of revolutions and their perceived levels of exertion to the primary investigator by a postal questionnaire once every two weeks, together with an overstrain questionnaire. Parents and their boys are informed by telephone when compliance is inadequate or when other problems (such as signs off overstrain: see adverse events) are identified and children are encouraged to resume the training or to adjust the training intensity.

#### Study outcomes

Study outcomes include measurements at the levels of body functions and structures, activities and participation as defined by the International Classification of Functioning, Disability and Health (ICF) [29]. Environmental characteristics (school type and home status) are assessed at baseline. Demographic variables (age, weight and arm span), the use of medication (such as corticosteroids), any co-interventions and daily amount of physical activity are registered during each session.

**Figure 4:**
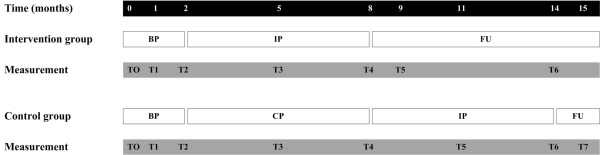
**Measurements study 1**. BP (Baseline period), IP (Intervention period), FU (Follow-up), CP (Control period)

The assessments of boys in the intervention group are conducted during the baseline period (T0: at baseline; T1: after 4 weeks; T2: after 8 weeks), the training period (T3: after 12 weeks; T4: after 24 weeks) and the follow-up (T5: 4 weeks after the end of the training; T6: 24 weeks after the end of the training). The assessments of boys in the control group are conducted during the baseline period (T0: at baseline; T1: after 4 weeks; T2: after 8 weeks), the control period (T3: after 12 weeks; T4: after 24 weeks), the subsequent training period (T5: after 12 weeks of training; T6: after 24 weeks of training) and during the follow-up (T7: 4 weeks after the end of the training). Assessments take place at the department of Rehabilitation of the Radboud University Nijmegen Medical Centre (T0, T2, T4, T6), but also at home (intervention group: T1, T3, T5; control group: T1, T3, T5, T7) to reduce the practical burden on the participants. All data are collected by the (trained) primary investigator (MJ). (figure 4)

##### Primary outcomes

Bicycle ergometry: Six-Minute Bicycle Test

Muscle endurance, in this study defined as 'the ability to sustain a power without the occurrence of peripheral fatigue', is assessed with an adjusted and modified bicycle ergometer test for both the lower and upper extremities. Bicycle ergometry has been used in many studies to examine the (an)aerobic performance in healthy children and children with disabilities, and several exercise protocols are available. However, these protocols are not feasible for boys with DMD, since large muscle groups are affected in DMD and initial load and subsequent increments of load are often too hard to sustain for these patients[7,30]. Muscle endurance is, therefore, examined with a recently developed motor-assisted (passive mode 1, no-load speed 7 RPM) Six-Minute Bicycle Test by which boys are instructed to cycle as fast as possible i.e. to make as many cycling revolutions as possible. Boys start with a test for their legs and, thereafter, perform the same (arm cranking) test with their arms.

The occurrence of muscle fatigue during bicycle ergometry is assessed objectively with bipolar surface electromyography (sEMG), since peripheral fatigue is reflected as an increase in the amplitude and a decrease in the median frequency of the sEMG signal[31,32]. These parameters were found to be responsive to changes in muscle endurance during an exercise program[33,34]. Electrodes are placed unilaterally (right leg, right arm) on the m. rectus femoris, m. vastus medialis, m. biceps femoris, m. tibialis anterior, m. biceps brachii, m. triceps brachii and m. deltoideus. Electrode placement procedures will follow the recommendations for Sensor and Sensor Placement Procedures for surface electromyography[35]. Data will be registered with an EFA system (Twente Medical Systems International). The occurrence of muscle fatigue is also assessed subjectively with the OMNI Scale for perceived exertion[27]. The OMNI Scale is reliable and valid over a wide range of dynamic exercise intensities in children[27,36].

Motor Function Measure

The MFM is a recently developed instrument to assess motor function in ambulant and non-ambulant patients with neuromuscular diseases (NMD) aged 6-62 years[37]. The MFM has shown excellent internal consistency and good to excellent intra- and interrater reliability in French neuromuscular patients[37]. Vuillerot et al.[38] showed that the MFM is able to measure changes in motor function over time in boys with DMD. An ongoing study examines validity and applicability of the MFM in rehabilitation institutes in the Netherlands.

##### Secondary outcomes

Table 2 shows an overview of the secondary study outcomes and their psychometric properties.

**Table 2:** Outcome measures and psychometric properties

Level	Study outcome	Measurement tool	Psychometric properties	Assessment
**Body structures and functions**	Muscle endurance	Six-Minute Bicycle Test*	Feasible for ambulant and non-ambulant boys with DMD (pilot study, unpublished data)	T0, T2, T5

	Joint mobility (PROM)	Goniometry[55] (knee ext*, ankle dfl*, shoulder abd*▪, elbow ext*▪, wrist ext▪, wrist radial and ulnar dev▪)	Standardized methods are feasible[56] and have good intra- and inter-rater reliability in DMD[57] Passive wrist radial deviation is correlated with functional hand activities[58] and lower extremity contractures are related to onset of wheelchair reliance in DMD[4]	All

	Muscle strength	Modified MRC[59] (hip ext*, knee ext* ankle dfl*, shoulder abd*▪, elbow ext*▪, wrist ext▪)	Moderate to good intra-rater reliability[59] and acceptable inter-rater reliability in DMD after a training session[60] Muscles with MRC grade 4 or 5 are difficult to measure with MMT, but muscles with MRC grade ≤3 are more difficult to measure with HHD[4]	All

	Muscle atrophy, intra-muscular fibrosis and fatty infiltration	Quantitative skeletal muscle ultrasonography (muscle thickness and echo intensity) [61]: RF, TA, BB, FF*▪	Good inter-rater agreement in children[62] High predictive values to discriminate between children with and without a NMD[63]	T2, T5, T6/T7*

	Bone density	Dexascan (femur and lumbar spine)*	Changes in bone mineral density can be detected with confidence in healthy boys ≥10 years after 6 months and in younger boys after 12 months[64], but a change in body shape may influence scan results[65]	Conventional protocol for each boy

	Incidence of fractures	Semi-structured interview*		All

**Activities**	Functional abilities	Motor Function Measure[37] (D1*, D2*, D3*▪)	Excellent internal consistency for the global scale and the subscales in NMD[37] Excellent to moderate intra- and inter-rater reliability in NMD[37] Good face validity, convergent validity and discriminant validity in NMD[37] Sufficiently sensitive to detect changes in the total score in DMD[66] Total score predicts loss of ambulation in DMD[67]	All

	Upper limb function	Action Research Arm Test[40,41] ▪	Excellent intra-rater, inter-rater and test-retest reliability in stroke patients[40,41] Highly correlated with the Fugl-Meyer score^47 ^and Functional Independence Measure^48 ^in stroke patients Suitable to detect changes over time in stroke patients[42]	All

	Functional abilities (grading)	Vignos* and Brooke Scale*▪ [56]	Good inter-rater and intra-rater reliability[57,57] and correlated with timed tests[46,68,69] in DMD	All

	Functional mobility	Functional Mobility Scale[70] *	A clinically feasible, valid and reliable tool in CP[70,71]	All

	Functional abilities (timed tests)	Timed and graded functional tests (and total GSGC score) [72]: walk 10 meters, climb 3 stairs, rise from the floor and rise from a chair*	Good to excellent intra- and inter-rater reliability in DMD[57,73] Sensitive to change in DMD: a small reduction in muscle force was accompanied by a large increase in time it takes to complete functional tests[74]	All (gait, stairs and chair only in the hospital)

	Finger dexterity	Nine-hole Peg Test[75] *▪	Moderately high test-retest reliability, high inter-rater agreement and adequate concurrent validity in school-age children[76]	All

	Hand function	Jebsen-Taylor Hand Function Test[77] ▪	Good test-retest reliability in DMD[58] Strongly correlated with muscle strength of the wrist extensors[58], radial deviation range of motion[58] and the Brooke scale[46] in DMD	T2, T4, T5

	Functional status	PEDI[78,79] (selfcare*▪ and mobility*)	Good inter-rater and test-retest reliability[80], content validity[79] and discriminative validity[81] in children with various diagnosis Feasible for evaluative purposes in CP[81,82]	T0, T2, T4, T6/T7*

	Perceived manual abilities	Abilhand[83] ▪ Abilhand-kids[84] *	The Rasch-derived Abilhand is moderately related to grip and key pinch strength, has good test-retest reliability and may be sensitive to change in stroke patients[85] The Abilhand-kids has good test-retest reliability and a higher independence in gross motor function is associated with a higher manual ability in CP[84]	T0, T2, T4, T6/T7*

	Quality of upper-limb motor function	Melbourne Assessment of Unilateral Upper Limb Function[86] (item 1,2,3,10,11 and16) extended with an upper limb motion analysis (Vicon Motion Systems) with 8 cameras▪	The Melbourne Assessment has moderate to high intra- and inter-rater reliability[87] and excellent construct validity in CP[88] A motion capture analysis system can measure task performance with an upper-limb orthosis[45], but soft tissue artefacts may negatively influence accuracy[49]	T2, T4

	Incidence and fear of falls	Semi-structured interview*		All

**Participation**	HRQoL	KIDSCREEN-52[89](child- and parental questionnaire)*▪	Acceptable levels of reliability and validity in children and adolescents[90] Children's most important in their lives generally map well to the items in KIDSCREEN[91]	T0, T2, T4, T6/T7*

**Demographic variables**	Weight and height	Body weight (kg)*▪, standing height* (cm) and arm-span*▪ (cm)		T0*, T2, T4, Y6/T7*

**Co-factors**	Co-interventions	Semi-structured interview*▪		All

	Physical activity	Semi-structured interview (according to the PAQ-C[92] and the 60-min MVPA measure[93])*▪		All

Symbols:* = study 1 'Dynamic leg and arm training', ▪ = study 2 'Functional training with arm support'

**Abbreviations**: DMD: Duchenne Muscular Dystrophy; PROM: Passive Range of Motion; ext: extension; dfl: dorsal flexion; abd: abduction; dev: deviation; MRC: Medical Research Council Scale; RF: m. Rectus Femoris; TA: m. Tibialis Anterior; BB: m. Biceps Brachii; FF: forearm flexors; NMD: Neuromuscular Disease; GSGC: Gait, Stairs, Gowers, Chair; PEDI: Pediatric Evaluation of Disability Inventory; CP: cerebral palsy; HRQoL: Health-Related Quality of Life; PAQ-C Physical Activity Questionnaire for older children; MVPA: Moderate-to-Vigorous Physical Activity

#### Adverse events

All adverse events will be recorded. The study will be terminated prematurely for an individual participant when training is excessive and remains excessive after a reduction of the training intensity. Signs for overstrain are: excessive muscle pain during the training, prolonged post-exercise muscle pain, a severely uncomfortable feeling during or after the training and extreme (muscle) fatigue (OMNI scale > 6).

#### Sample size

The average rate of decline (mean and standard deviation (SD)) of muscle endurance and functional abilities in boys with DMD, as assessed with bicycle ergometry and the Motor Function Measure (MFM), is unknown. Therefore, no sample size can be calculated for these primary outcomes. However, it is anticipated that multiple baseline measurements will increase the chance of finding statistically significant changes in the selected outcome measures. For reasons of convenience, and because this RCT should be regarded as a first explorative study, we decided to include 20 boys in the intervention group and 10 boys in the control group.

#### Analysis

A two-way analysis of variance (ANOVA) will be used to examine the effects of dynamic leg and arm training on the primary outcomes. Secondary outcomes will be treated similarly or by non-parametric analyses dependent on the type of outcome (interval or ordinal). Data will be analyzed with SPSS version 16.0 and an alpha level of 0.05 will be used to decide on statistical significance.

### Study 2 Functional training with arm support

#### Design

Study 2 has an observational one-group design using repeated measurements, because we anticipate that it will not be possible to recruit a sufficient number of boys for an RCT in the Netherlands. This problem is related to the fact that boys with DMD who have been confined to a wheelchair already for several years often experience many barriers, such as a lack of time due to therapy obligations and homework. It is also virtually impossible for them to do without their electric wheelchair for a couple of days to build up the required arm support for this study. In addition, the number of boys at this stage in the course of DMD is low (i.e. an estimated 120 boys in the Netherlands) and, it is expected that some of these boys already use an arm support, which is an exclusion criteria for participation.

#### Participants

**Figure 5:**
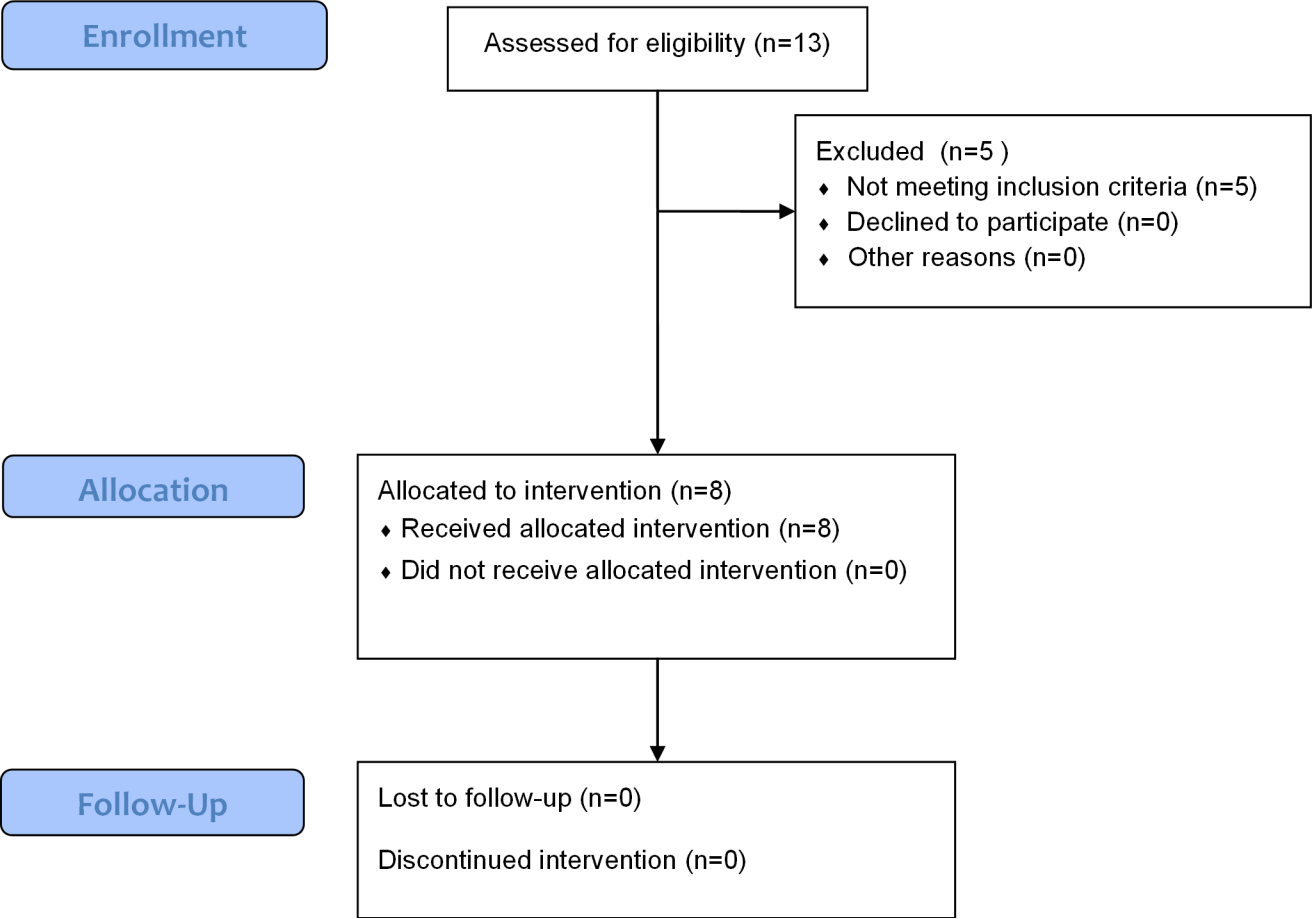
**Progress study 2 Functional training with arm support**. This flowchart is a preliminary version. Final numbers may change depending on eligibility as recruitment is not yet complete.

Ten wheelchair-dependent boys with a DNA-established diagnosis of DMD will be recruited in the same way as the participants of study 1 (figure 5). Inclusion and exclusion criteria are described in table 3. Participants will receive written information and will be visited by the primary investigator at home for providing further information. Both parents and participants will need to give written informed consent. The study protocol was approved by the Medical Ethics Committee Arnhem-Nijmegen, the Netherlands.

**Table 3:** Inclusion and exclusion criteria study 2

Inclusion criteria	Exclusion criteria
A DNA-established diagnosis of DMD	Other disabling diseases influencing mobility
Boys who have been wheelchair-dependent for a few years (approximately 2-5 years after they stopped walking)	Boys who are able to stand
	Boys >20 years old
Boys who have problems with reaching and lifting movements with their arms, and: -are unable to touch the top of their head (at least with one hand) -are still able to use their hands for some daily activities	Boys who already use an arm support

#### Intervention

**Figure 6:**
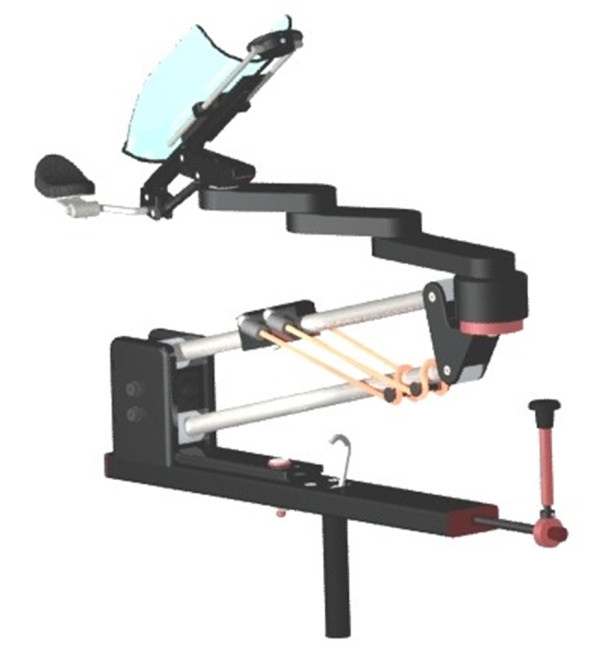
**Dynamic arm support Top/Help**. Focal Meditech BV, Tilburg, the Netherlands (www.focalmeditech.nl)

The six-months physical training consists of 1) a computer-assisted training and 2) a functional training of the non-dominant arm and hand (i.e. the hand one does not use for writing) with a mechanical or an electrical arm support (Dynamic Arm Support Top/Help, Focal, Tilburg, the Netherlands) (figure 6). Boys who are unable to touch their nose with a mechanical arm support (due to insufficient muscle strength) will receive an electrical arm support. The arm support transposes shoulder movements into movements of the forearm and hand, which increases functional abilities and joint range of motion in boys with DMD. During the first baseline measurement (T0), boys will try to fit the arm support under supervision of an experienced representative of Focal (the manufacturer of the TOP arm support) and the primary investigator. Training of the non-dominant arm is additional to any regular therapy. All co-interventions will be registered.

##### Computer-assisted training

Boys play "Furballhunt" (developed by Roessingh Research and Development B.V. (M.A. Jannink), Enschede, the Netherlands) to practice targeted forward and sideward reaching movements (ipsi- and contra-lateral) as well as lifting movements during five days per week. Furballhunt is a computer game that was originally developed to improve arm function in children with cerebral palsy using a virtual reality environment. Furballhunt is based on motion capture technology and uses a webcam (in this study: Logitech QuickCam E3500) to detect gross shoulder and elbow movements. Birds ("Furballs") fly from their bird-house to a tree branch while the boy holds his hand in front of his navel. Boys have to touch the Furball when it sits down on a tree branch. The faster boys chase away Furballs, the higher their score. A virtual reality computer game is considered to be a motivational tool for training in children[39].

Boys play five games of Furballhunt (30 Furballs per game, 30 sec of rest between games) per day at home. The number of tree branches (3), game speed (5/10), the number of Furballs (30) and the sequence of games are standardized. Training intensity is low to moderate, which means that boys are allowed to perceive their exertion as 'getting a little tired' or 'getting more tired' as assessed with the OMNI Scale[27]. Intensity is adjusted to the physical abilities of the boy by varying the position of the tree branches, which represent the targets to move to. Boys are instructed to move their arm over the full range of motion, which may cause a feeling of stretch but not pain.

Boys report their training frequency in a diary and complete an overstrain questionnaire once every two weeks. Parents and their boys are informed by telephone when compliance is inadequate or when other problems (such as signs of overstrain: see adverse events) are identified. Boys are encouraged to resume the training and to adjust the training intensity, when appropriate. The date, training time and reaction times are saved on the computer, so that compliance and performance can be checked afterwards by the primary investigator.

##### Functional training

Boys should eat at least one meal with the arm support twice every week. In addition, they are instructed "to use the arm support as much as possible every day". Boys keep a written record of all activities they perform with the arm support in a three-day diary once every two weeks (two weekdays, one weekend day).

#### Study outcomes

Study outcomes include measurements at the levels of body functions and structures, activities and participation of the ICF[29]. Environmental characteristics (school type and home status) are assessed at baseline. Demographic variables (age, weight, arm span), the use of medication (such as corticosteroids) and any co-interventions are registered during each session.

**Figure 7:**
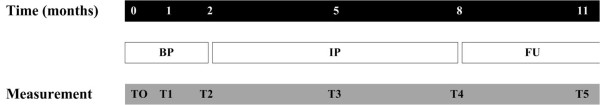
**Measurements study 2**. BP (Baseline period), IP (Intervention period), FU (Follow-up)

After a two-month period for baseline measurements (T0: at baseline; T1: after 4 weeks; T2: after eight weeks) to obtain information about the stability of the course of DMD, training with the arm support takes place for six months. Assessments take place after 12 weeks (T3) and 24 weeks (T4) training. Finally, one extra measurement will be done after 12 more weeks (T5) to evaluate to what extent the possible effects of training have lasted. Data is collected by the primary investigator (MJ) at the department of Rehabilitation of the Radboud University Nijmegen Medical Centre (T2, T4), but also at home (T0, T1, T3, T5) to reduce the practical burden on the participants. (figure 7).

##### Primary outcome

Action Research Arm Test (ARAT)

As no suitable measure of arm motor function has been validated for children with NMD, the functional abilities of the upper extremity (reaching, lifting and manipulating) are assessed bilaterally with the Action Research Arm Test (ARAT)[40]. The ARAT is a standardized tool for the assessment of arm motor function and capacity (both distally and proximally) and consists of 19 items in four dimensions: grasp, grip, pinch and gross movements. The ARAT has shown to be reliable[40,41], valid[40-42] and suitable to detect changes over time[42] in stroke patients. The ARAT is performed and scored following standardized procedures as described by Yazbatiran[41]. The non-dominant arm is assessed both with and without arm support.

##### Secondary outcomes

Table 2 shows an overview of the secondary study outcomes and their psychometric properties. Both the dominant and non-dominant arms are assessed. The non-dominant arm is assessed both with and without arm support.

#### Adverse events

All adverse events will be recorded. The study will be terminated prematurely for an individual participant when training is excessive and remains excessive after a reduction of the training intensity. Signs for overstrain are: excessive muscle pain during the training, prolonged post-exercise muscle pain, a severely uncomfortable feeling during or after the training and extreme (muscle) fatigue (OMNI scale > 6).

#### Sample size

This is a first explorative study that will give an indication of the effect of functional training with arm support on the functional abilities of the upper extremity in boys with DMD. In this perspective, no realistic power calculation can be made. By using a design with multiple measurements at baseline we anticipate to find statistically significant effects of the proposed therapeutic intervention in ten boys with DMD.

#### Analysis

A one-way analysis of variance (ANOVA) of time will be used to examine the effects of functional arm training with arm support on the primary outcome (ARAT). Secondary outcomes will be treated similarly or by non-parametric analyses dependent on the type of outcome (interval or ordinal). Data will be analyzed with SPSS version 16.0 and an alpha level of 0.05 will be used to decide on statistical significance.

## Discussion

This paper presents the research questions, hypotheses and methods of the No Use is Disuse (NUD) study in the Netherlands. The NUD study examines the effects of low-intensity dynamic physical training in boys at different stages in the course of Duchenne Muscular Dystrophy (DMD). It is expected that these dynamic exercises will be beneficial and will not impose a risk of muscle or other injury to the boys. This hypothesis is based on the (limited amount of) evidence that is available about physical training in children with neuromuscular diseases (NMD). In addition, the study was designed with the extensive input from several experts in the field of NMD in children (1 pediatric physiotherapist, 2 occupational therapists, 1 rehabilitation physician, 1 exercise physiologist, 1 neurologist, 1 clinical neurophysiologist and 1 epidemiologist). We will discuss the most relevant issues and decisions concerning 1) training (intensity), 2) outcome measures and 3) strategies to optimize therapy compliance.

### Training (intensity)

Boys in both studies are instructed to train five days per week during 24 weeks. Although this training frequency is high, it was chosen to elicit as much 'daily physical activity' as possible in every boy. Many boys with DMD, especially those who are confined to a wheelchair, have a sedentary lifestyle[28] and do not engage in 60 min of moderate daily physical activity as recommended by the WHO for children to reduce their risk of chronic diseases[43]. On the other hand, as boys with DMD already have a chronic disease, their physical limitations have to be taken into account to prevent exercise-induced physical deterioration[44]. Nevertheless, the level of daily low-intensity physical activity as prescribed in this study is thought to be safe and effective in reducing disuse atrophy and excessive functional loss.

Although training-induced improvements of muscle functions can normally be expected after six weeks of physical training[26], we have chosen a training period of 24 weeks. This relatively long training period is considered necessary, as the disease progression is relatively slow, and the expected training effects over time probably small. We also assume that if boys are able to adhere to the training regime during these 24 weeks, it will be feasible for them to incorporate physical training in daily life afterwards.

Boys in study 2 train reaching and lifting movements, since reaching is one of the tasks with a high priority in potential users of an arm support[45]. Boys train their non-dominant arm as this arm is used less than the dominant arm in functional acitivities[46]. Therefore, training effects are expected to be larger for the non-dominant arm. Nevertheless, it may be difficult for boys to perform activities (like eating and scratching) with their non-dominant arm.

### Study outcomes

In this study, reliable functional scales are used, such as the MFM, as well as timed tests that have clinically meaningful endpoints[47] and that are sufficiently sensitive to detect therapy related gains[48]. Indeed, outcome measures should be reliable and sensitive to allow for an adequate power in trials with a relatively small sample size. As no suitable ergometry test or arm motor function test is available for (non-ambulant) boys with NMD, a Six-Minute Bicycle test and the ARAT have been selected as primary outcomes. Pilot studies showed that the applied Six-Minute Bicycle test was feasible for both ambulant and recently wheelchair-dependent boys with DMD. The ARAT proved to be useful in a wheelchair-dependent boy with DMD as well (unpublished data). In addition, we used an upper extremity protocol to quantitatively measure arm motor function[49] with an eight camera motion analysis system (Vicon Motion Systems, Ltd, Oxford, UK). It has been shown that such a system can accurately measure task performance with an upper-limb orthosis[45]. Arm motor function has never been measured quantitatively before in boys with DMD, which may be of value because of its continuous properties and presumably greater sensitivity to change[50].

### Strategies to increase therapy compliance

From a previous study, it can be concluded that home-based cycling programs for children are feasible and show good adherence rates[51]. A home-based program allows boys to train at times that are convenient for them and reduces travel time. However, a home-based program requires great discipline from boys and their families, and several aspects need attention to optimize therapy compliance. One of these aspects, according to the Social Cognitive Theory[52], is that the social environment (e.g. family and school) is essential for a change in health behavior. Therefore, to optimize therapy compliance, parents are asked to stimulate their boys by reminding them to perform their exercises. In addition, siblings can be role models by being physically active themselves. Other strategies that are used to enhance behavioral change and optimize therapy compliance are specific goal setting[53] and reduction of perceived barriers[54]. For this reason, as described previously, all boys sign a training contract including an agreement on the training moments. The barriers that boys or their parents might perceive are discussed beforehand. In addition, the boys in study 1 are recommended to train while watching the television to make the training more pleasurable.

### Expected products

The NUD study is the first clinical trial that examines the effects of low-intensity physical training on muscle endurance and functional abilities in boys with DMD. It will be a start to fill the current gap in our knowledge about the efficacy of physical training in these boys and will increase our insight into what type of physical training should be recommended. Although the NUD study focuses on children with DMD, the results may be (partly) applicable to other neuromuscular disorders in childhood. Results of the NUD study are expected by the end of 2010.

## Competing interests

The authors declare that they have no competing interests.

## Authors' contributions

IJMG and NA handled funding and designed the first draft of the study protocol. ACHG was responsible for supervision of the project. All authors developed and wrote the final study protocol (drafted by MJ, critically revised by IJMG, NA and ACHG) and gave approval of the version to be published.

## Pre-publication history

The pre-publication history for this paper can be accessed here:

http://www.biomedcentral.com/1471-2431/10/55/prepub
